# Migrating Birds and Tickborne Encephalitis Virus

**DOI:** 10.3201/eid1308.061416

**Published:** 2007-08

**Authors:** Jonas Waldenström, Åke Lundkvist, Kerstin I. Falk, Ulf Garpmo, Sven Bergström, Gunnel Lindegren, Anders Sjöstedt, Hans Mejlon, Thord Fransson, Paul D. Haemig, Björn Olsen

**Affiliations:** * Kalmar University, Kalmar, Sweden; † Lund University, Lund, Sweden; ‡ Karolinska Institute, Solan, Sweden; § Umeå University, Umeå, Sweden; ¶ Uppsala University, Uppsala, Sweden; # Swedish Museum of Natural History, Stockholm, Sweden

**Keywords:** Migratory birds, ticks, tickborne encephalitis virus, TBE, TBEV, long-distance dispersal, biogeography, dispatch

## Abstract

During spring and autumn 2001, we screened 13,260 migrating birds at Ottenby Bird Observatory, Sweden, and found 3.4% were infested with ticks. Four birds, each a different passerine species, carried tickborne encephalitis virus (TBEV)–infected ticks (*Ixodes ricinus*). Migrating birds may play a role in the geographic dispersal of TBEV-infected ticks.

Tickborne encephalitis is a viral zoonotic disease caused by the tickborne encephalitis flavivirus (TBEV). There are 3 subtypes of TBEV: the European subtype (TBEV-Eu, transmitted by *Ixodes ricinus* ticks) and the Siberian and Far-Eastern subtypes (TBEV-Sib and TBEV-FE, transmitted by *I. persulcatus* ticks) (*1*–*3*). Geographic distribution of TBEV subtypes largely follows that of their tick hosts: *I. ricinus* (Europe) and *I. persulcatus* (from Far East to the Baltic countries) (*4*). In Latvia and Estonia, the distribution of both tick species overlaps, and all 3 TBEV subtypes cocirculate in Latvia (*3*). Thus, a range expansion of a tick species could result in spreading a TBEV subtype to new areas.

Small rodents are thought to be the main amplifying hosts, although wild ungulates contribute indirectly by providing blood meals for adult ticks, thereby maintaining the vector populations necessary for virus transmission. In addition to mammals, *I. ricinus* ticks take blood meals from birds, which has led to speculation that birds could disperse TBEV-infected ticks during migration and start new TBE foci. In this study, we document the occurrence of TBEV-infected ticks in migrating birds.

## The Study

Fieldwork was conducted during 2001 at Ottenby Bird Observatory, located on the southernmost tip of Öland, a large island off the southeast coast of Sweden (56° 12′ N, 16° 24′ E; Figure). Throughout spring (March 25–June 15) and autumn (July 1–November 15) migration, observatory personnel captured and screened birds for ticks, except during 8 days when an excessive number of trapped birds made complete monitoring impossible. Each captured bird was identified by species and age and was banded. For bird species with TBEV-infected ticks, local banding and recovery records from 1946 to the present were used to determine recruitment and wintering areas.

**Figure:**
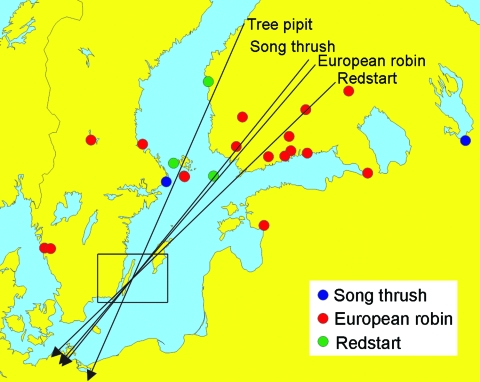
Autumn migration directions (arrows) of tree pipits (*Anthus trivialis*)*,* robins (*Erithacus rubecula*), redstarts (*Phoenicurus phoenicurus*)*,* and song thrushes (*Turdus philomelos*) banded in southeastern Sweden (area indicated by a square) and recovered within 60 days. Directions: Tree pipit, 203.6º, mean vector length = 0.993, n = 10; robin, 220.5º, mean vector length = 0.928, n = 293; redstart, 225.9º, mean vector length = 0.975, n = 52; and song thrush, 218.8º, mean vector length = 0.947, n = 117. Recovery sites of birds banded in southeastern Sweden and reported from areas north of the banding sites in a following year are also shown as indicated in the legend (no recovery from breeding areas is available for tree pipit).

Tick screening comprised rapid visual assessment for the presence of any ticks on bare body parts, especially around the eyes and beak of each bird. All ticks were removed by forceps, placed separately into snap-lid tubes, frozen and stored at –70^ο^C, and then analyzed with a dissecting microscope to identify species and development stage.

A Puregene RNA isolation protocol adopted for 100–10,000 cells (Gentra Systems, Minneapolis, MN, USA) individually homogenized each tick and extracted RNA, according to the manufacturer’s instructions. The RNA pellet was resolved in 25 µL DNA hydration buffer and stored at –70°C until further analysis.

Samples were pooled 10 by 10 (5 µL from each individual extract) and analyzed by a nested reverse transcription (RT)-PCR targeting the 5′-terminal noncoding region (*5*) for the initial detection of TBEV RNA. Briefly, the RT-PCR was performed in 25-µL reaction volumes containing 1× EZ buffer, 0.3 mmol of each deoxyribonucleotide (dNTP), 2.5 U rTth DNA polymerase, 2.5 mmol Mn(OAc)_2_ (all reagents provided from Perkin Elmer, Branchburg, NJ, USA), 25 pmol of each primer (Pp1 and Pm1), 25 U Rnasine (Gibco, Paisley, Scotland, UK), and the target viral RNA. The reaction was performed in a GeneAmp 9700 thermal cycler (Applied Biosystems, Foster City, CA, USA) programmed to incubate 45 min at 60°C for RT and 2 min at 94°C for denaturation as initial steps, followed by 40 cycles of 30 s at 94°C and 30 s at 66°C. The final extension was for 5 min at 66°C. Negative and positive controls were included in each PCR run.

A second amplification step was conducted with 2 µL of the first amplification products. The total reaction volume of 25 µL included 1× PCR buffer II, 1.5 mmol MgCl_2_, 0.2 mmol each of dNTP, 0.625 U AmpliTaq Gold polymerase (Perkin Elmer), and 25 pmol of each internal primer (Pp2 and Pm2). After a pre-incubation step of 9 min at 95°C, the reaction was continued by 30 cycles of 15 s at 94°C and 30 s at 65°C and ended with an elongation step of 10 min at 72°C. Samples from positive pools were rerun using individual samples with the nested PCR described above.

During the study period, 1,155 ticks were collected from 447 (3.4%) of 13,260 screened birds (Table). Nearly all ticks (1,130) were reliably identified as *I. ricinus.* Seven nymphs showed characters resembling *I. lividus,* but these and 19 other ticks were rather poorly preserved, making identification uncertain. Frequencies of the various tick life stages were as follows: larvae (53.4%), nymphs (45.1%), and adults (0.6%). The mean infestation rate (0.086 immature ticks per examined bird, 2.6 immature ticks per infested bird) was unevenly distributed among bird species, with tick infestation in only 37 of >100 investigated species.

**Table:** Bird species infested with ticks during the spring and autumn migration periods

Scientific name	Common name	Spring				Autumn		
		No. infested	No. ticks	Infestation rate (ticks/infested bird)		No. infested	No. ticks	Infestation rate (ticks/infested bird)
*Accipiter nisus*	Eurasian sparrowhawk	–	–	–		1	2	2.0
*Acrocephalus palustris*	Marsh warbler	1	1	1.0		–	–	–
*Acrocephalus scirpaceus*	European reed warbler	–	–	–		1	1	1.0
*Alauda arvensis*	Eurasian skylark	–	–	–		1	6	6.0
*Anthus trivialis*	Tree pipit	1	1	1.0		10	28	2.8
*Carduelis cannabina*	Common linnet	1	1	1.0		–	–	–
*Carduelis chloris*	European greenfinch	6	6	1.0		1	1	1.0
*Carduelis flammea*	Common redpoll	–	–	–		1	1	1.0
*Carduelis spinus*	Eurasian siskin	1	1	1.0		–	–	–
*Carpodacus erythrinus*	Common rosefinch	–	–	–		1	1	1.0
*Certhia familiaris*	Eurasian treecreeper	–	–	–		2	2	1.0
*Dendrocopos major*	Great spotted woodpecker	–	–	–		1	8	8.0
*Emberiza schoeniclus*	Common reed bunting	–	–	–		1	1	1.0
*Erithacus rubecula*	European robin	35	58	1.7		153	404	2.6
*Fringilla coelebs*	Common chaffinch	1	1	1.0		1	8	8.0
*Hippolais icterina*	Icterine warbler	–	–	–		2	15	7.5
*Lanius collurio*	Red-backed shrike	–	–	–		2	7	3.5
*Luscinia luscinia*	Thrush nightingale	2	5	2.5		2	4	2.0
*Luscinia svecica*	Bluethroat	3	5	1.7		2	3	1.5
*Parus caeruleus*	Eurasian blue tit	2	3	1.5		4	11	2.8
*Parus major*	Great tit	2	2	1.0		18	34	1.9
*Phoenicurus phoenicurus*	Common redstart	3	6	2.0		9	18	2.0
*Phylloscopus sibilatrix*	Wood warbler	–	–	–		1	1	1.0
*Phylloscopus trochilus*	Willow warbler	3	3	1.0		16	18	1.1
*Prunella modularis*	Dunnock	2	6	3.0		2	3	1.5
*Pyrrhula pyrrhula*	Eurasian bullfinch	–	–	–		5	8	1.6
*Regulus regulus*	Goldcrest	–	–	–		1	1	1.0
*Sturnus vulgaris*	Common starling	–	–	–		10	19	1.9
*Sylvia atricapilla*	Blackcap	1	2	2.0		6	6	1.0
*Sylvia borin*	Garden warbler	–	–	–		1	1	1.0
*Sylvia communis*	Common whitethroat	6	13	2.2		12	33	2.8
*Sylvia curruca*	Lesser whitethroat	2	4	2.0		6	7	1.2
*Sylvia nisoria*	Barred warbler	2	3	1.5		1	1	1.0
*Troglodytes troglodytes*	Winter wren	7	16	2.3		11	19	1.7
*Turdus iliacus*	Redwing	8	17	2.1		2	5	2.5
*Turdus merula*	Common blackbird	24	86	3.6		20	89	4.4
*Turdus philomelos*	Song thrush	7	12	1.7		18	131	7.3
*Turdus pilaris*	Fieldfare	1	4	4.0		2	2	1.0
	Total	121	256			326	899	

Ground-foraging birds carried ≈80% of all detected ticks and made up 71.3% of all infested birds (Table). A few ticks were also found on granivorous bird species, e.g., siskins, finches, sparrows, and some insectivorous songbirds, particularly among *Sylvia* and *Acrocephalus* warblers that forage in reed beds or dense stands of herbaceous plants (Table). The number of detected ticks per infested bird was usually in the range of 1–5 ticks, but 2 birds, a song thrush (*Turdus philomelos*) and a European robin (*Erithacus rubecula),* carried 41 and 39 ticks, respectively.

After initial screening of pools and rerunning individual samples from PCR-positive pools, we detected 6 TBEV-positive samples: 4 tick nymphs and 2 larvae. One larva was collected from a juvenile tree pipit (*Anthus trivialis)*, 1 nymph each from a song thrush and juvenile redstart (*Phoenicurus phoenicurus*), and 2 nymphs and 1 larva from a juvenile European robin. All TBEV-infected ticks were collected from birds during the autumn migration. Despite repeated trials, we were unable to obtain readable sequence data from the positive samples and could not identify the TBEV strains by subtype.

## Conclusions

Our study found that some ticks attached to birds carried TBEV. However, the frequency of TBEV among such ticks was less than the frequency of *Borrelia burgdorferi* senso lato from similar datasets *(**6**–**8*). Analyses of banding recovery data for the 4 bird species with TBEV-infected ticks indicate an eastern recruitment area coinciding with TBE-endemic areas in Fennoscandia and western Russia (Figure).

TBEV has been isolated, or serologically indicated, from several bird species, especially anatids and gallinaceous birds, and most often from Eastern Europe or Russia *(**9**)*. However, little is known about the capability of birds to function as reservoirs of TBEV, and small rodents remain the most important reservoirs of the virus. The fact that we found 2 *I. ricinus* larvae infected with TBEV could indicate that these birds may be reservoirs, because these larvae did not feed before attaching themselves to the birds. However, nonviremic transmission between ticks cofeeding on the same host has been shown to occur with TBEV *(**10*) and other arboviruses *(**11**,**12**)*, and we did not look for viremia in the tick-infested birds.

The migration of birds through Scandinavia during spring and fall involves several hundred million birds. Although the tick infestation rate per bird was not great in our study, and TBEV-infected ticks were only a small fraction of all ticks, the vast numbers of migrating birds do increase the probabilities for geographic spread of ticks and TBEV, in particular for TBEV-Eu, because *I. ricinus* predominated in our sample. Our data add to the growing body of evidence showing that migratory birds can disperse ticks infected with medically important pathogens *(**6**,**7**,**10**,**13**)*.
